# Growth Patterns in Shwachman-Diamond Syndrome: Findings from the North American Shwachman-Diamond Syndrome Registry

**DOI:** 10.1016/j.jpeds.2025.114780

**Published:** 2025-08-14

**Authors:** Lois Schwarz, Leah Cheng, Sarah Steltz, Diana Schwarz, Elizabeth Korn, Sara Loveless, Richard Cooper, Claire Dusa, Lindsey Hornung, Jonathan C. Howell, Akiko Shimamura, Kasiani C. Myers, Jane Koo

**Affiliations:** 1 Division of Pediatric Hematology/Oncology, Dana-Farber/Boston Children’s Cancer and Blood Disorders Center, Department of Pediatrics, Harvard Medical School, Boston, MA;; 2 Clinical Research Operations Center, Boston Children’s Hospital, Boston, MA;; 3 Division of Bone Marrow Transplantation and Immune Deficiency, Cincinnati Children’s Hospital Medical Center, Department of Pediatrics, University of Cincinnati, Cincinnati, OH;; 4 Division of Biostatistics & Epidemiology, Cincinnati Children’s Hospital Medical Center, Cincinnati, OH;; 5 Division of Endocrinology, Cincinnati Children’s Hospital Medical Center, Department of Pediatrics, University of Cincinnati, Cincinnati, OH

## Abstract

**Objective:**

To characterize growth patterns in individuals with Shwachman-Diamond syndrome (SDS) in North America by generating SDS-specific growth curves and assessing the impact of hematopoietic stem cell transplant (HSCT) and growth hormone (GH) on growth.

**Study design:**

We conducted a retrospective cohort study of 127 subjects with confirmed biallelic Shwachman-Bodian-Diamond syndrome mutations enrolled on the North American SDS Registry. Height-for-age, weight-for-age, and body mass index (BMI)-for-age Z-scores were analyzed and compared with Centers for Disease Control and Prevention and World Health Organization reference populations. The effects of HSCT and GH therapy on growth velocity and final height outcomes were assessed.

**Results:**

Individuals with SDS demonstrated significantly lower height-for-age Z-scores, with median adult height being approximately 7 cm shorter in females and 11 cm shorter in males compared with Centers for Disease Control and Prevention standards. Although weight-for-age was diminished, BMI-for-age trajectories were preserved. HSCT significantly reduced height-for-age Z-scores slope, and in a small subsample, GH therapy showed limited efficacy in improving final height.

**Conclusions:**

This study of SDS across a diverse North American sample extends available growth data for this rare condition, confirming persistent short stature but normal BMI for age in SDS. HSCT was associated with a reduction in growth. No significant change in height velocity was observed with GH therapy. These data will inform clinical growth evaluation in SDS patients.

Shwachman-Diamond syndrome (SDS) is a rare, autosomal-recessive inherited bone marrow failure syndrome with an estimated incidence of 1 in 168,000 individuals.^1^ SDS is a disorder of ribosomal biogenesis and approximately 90% of patients with SDS have biallelic pathogenic variants in the Shwachman-Bodian-Diamond syndrome (SBDS) located on chromosome 7q11.^2^ Rare pathogenic variants in 3 other genes have been identified to cause similar phenotypes including DNAJC21, EFL1, and SRP54.^3^ SDS is classically characterized by neutropenia, exocrine pancreatic insufficiency, a predisposition toward progressive bone marrow failure, and elevated risk of hematologic malignancies and varying degrees of cognitive impairment.^4–6^ Hematopoietic stem cell transplantation (HSCT) remains the only curative therapy option for patients with SDS who develop bone marrow failure or transform to myeloid malignancy.^7^ Many different body systems can also be affected in individuals with SDS, including the endocrine, skeletal, immune, and gastrointestinal systems, although not all of these are affected simultaneously in all patients.^8–10^

Growth issues in SDS are likely due to a combination of nutrition, endocrine-related factors, and intrinsic effects of ribosomal impairment. Individuals with SDS can have difficulty absorbing nutrients due to exocrine pancreatic insufficiency, requiring specialized feeding strategies and pancreatic enzyme replacement therapy. In SDS, exocrine pancreatic insufficiency results from pancreatic acinar cell atrophy and adipocyte replacement without appreciable inflammation. The clinical phenotype of pancreatic dysfunction in patients with biallelic SBDS mutations present with pancreatic insufficiency is variable. However, 40%−60% of patients who begin with pancreatic insufficiency early on in life eventually become pancreatic sufficient over time.^11^ After diagnosis and the start of an appropriate pancreatic enzyme replacement and fat-soluble vitamin supplements, the growth rate is restored to a normal level in most of the children with SDS, although it consistently remains below the third percentile for height and weight.^12^

Endocrine factors contributing to short stature include congenital or acquired hormonal deficiencies inherent to SDS or due to chemotherapy exposure with HSCT procedures. The incidence of hormone (growth hormone [GH]) deficiency in individuals with SDS is variable.^13–15^ In a small cohort of patients with SDS (n = 6), the height standard deviation in patients with SDS significantly improved in the first 2 years of GH therapy compared with baseline. Growth velocity also improved significantly during the first year of GH treatment and remained higher than baseline for the second year.^16^ However, there are currently no studies that have described the impact of HSCT procedures on the growth patterns in individuals with SDS.

Recognizing the unique growth patterns observed in children and adolescents with SDS, Cipolli et al published a series of growth charts using anthropometric data from the Italian National Registry of SDS from individuals aged 0 to 18 years.^12,17^ The growth curves from this Italian SDS cohort were compared with the Cacciari percentiles^18^ for individuals aged 2 to 18 years, while the World Health Organization (WHO) reference ranges^19^ were used for individuals aged 0 to 2 years. However, the use of Cacciari percentiles may be less generalizable to populations outside Italy, as these references were derived from an Italian dataset. Although the Italian studies report a median age of menarche in females with SDS that is comparable to the general population (12 years), their focus on individuals aged less than 18 years may limit the ability to capture the full range of growth outcomes, particularly in those with delayed puberty or other growth-modifying factors. Furthermore, this group did not differentiate the impact of patients receiving GH therapy or those who had undergone HSCT, both of which could significantly influence growth trajectories.

Given these limitations, our study aims to advance our understanding of the growth patterns in individuals with SDS by characterizing a diverse cohort. We analyzed extended growth trajectories beyond 18 years of age in patients with SDS. In addition, we assessed the influence of GH therapy and HSCT on longitudinal growth outcomes. By expanding on prior studies, this work provides comprehensive, disease-specific growth references and evaluates key treatment effects, offering clinically relevant insights to guide management of growth in individuals with SDS.

## Methods

### Patient Sample

We performed a retrospective cohort study using data extracted from medical records of patients with genetically confirmed biallelic mutations in the SBDS gene consented to the North American SDS Registry. This study was approved by an ethics committee through Cincinnati Children’s Hospital Medical Center and Boston Children’s Hospital/Dana Farber Cancer Institute Institutional Review Boards. Anthropometric data from ages 0 to 20 years were extracted from patients’ medical records when patients were evaluated in outpatient clinic visits for routine follow-up. Any visits with anthropometric data for sick visits were excluded. We generated height, weight, and body mass index (BMI)-specific growth curves and fitted to established reference percentiles. SDS-specific percentiles were compared with WHO standards for patients aged 0 to 2 years and Centers for Disease Control and Prevention (CDC) references for patients aged 2 to 20 years. For the standard growth curves, we excluded any height and weight measurements taken at the time of or after HSCT or initiation of GH treatment. A separate analysis was performed in a subset of patients to evaluate the impact of HSCT and GH therapy on growth.

### Statistical Analysis and Generation of Growth Curves

Data were analyzed using SAS, version 9.4 (SAS Institute, Cary, NC). Due to skewed distributions or small numbers, continuous data were summarized as medians with interquartile ranges (interquartile range [IQR]: 25th-75th percentiles), while categorical data were summarized as frequency counts and percentages. Wilcoxon signed-rank tests were used to examine whether median SDS height-for-age Z-scores (HAZ) values differed from the expected value of 0. Generalized linear mixed models with random effects (to account for multiple measures longitudinally by the same subject) were used to analyze anthropometric measures and Z-scores over time. Growth curves were plotted using the reference percentiles from the CDC and WHO using Lambda Mu Sigma value calculations for percentiles overlayed with the fifth, 50th, and 95th percentiles from the SDS data for age and sex. A *P* value < .05 was considered statistically significant.

## Results

### Patient Cohort

This study included 127 patients (59% males and 41% females) with genetically defined SDS due to biallelic SBDS mutations (Table I). The most frequent combination of mutations in the SBDS gene was the c.258+2T>C splicing variant with the c.183–184TA>CT nonsense mutation, which was observed in 78 (61.4%) patients. In total, we had 2446 measurement observations for all patients aged between 0 and 20 years (on average 19.3 ± 17.8 observations per patient). Median age at diagnosis of SDS was 2.4 years (IQR: 0.6–7.7 years). The majority of patients (84/107, 78.5%) were born at a gestational age ≥ 37 weeks (full-term). Median birth weight for full-term infants was 2.9 kg (IQR: 2.5–3.3 kg) and median birth length was 19.0 inches (IQR: 18.0–19.4 inches).

As expected, 94% (118/126) of patients had a history of exocrine pancreatic insufficiency and of whom almost all received pancreatic enzyme replacement therapy (117/125, 94%). Twelve patients (10%) had received GH therapy. Indication for starting GH replacement was mixed, with 5 patients (42%) requiring GH for laboratory testing verified GH deficiency and 5 patients (42%) started on GH replacement for short stature. Thirty-seven patients (29%) had received HSCT. Patients underwent HSCT at a median age of 10.7 years (IQR: 5.7–20.8 years), with the majority of patients receiving HSCT for bone marrow failure (n = 14, 38%). Fifteen patients required HSCT for acute myeloid leukemia (n = 4, 11%) and myelodysplastic syndrome (n = 10, 27%). The majority of patients received reduced-intensity conditioning (n = 23, 62%) and only one patient (2.7%) received total body irradiation as part of their conditioning regimen.

### SDS Growth Curves for Ages 2–20 Years

**Figure 1.**
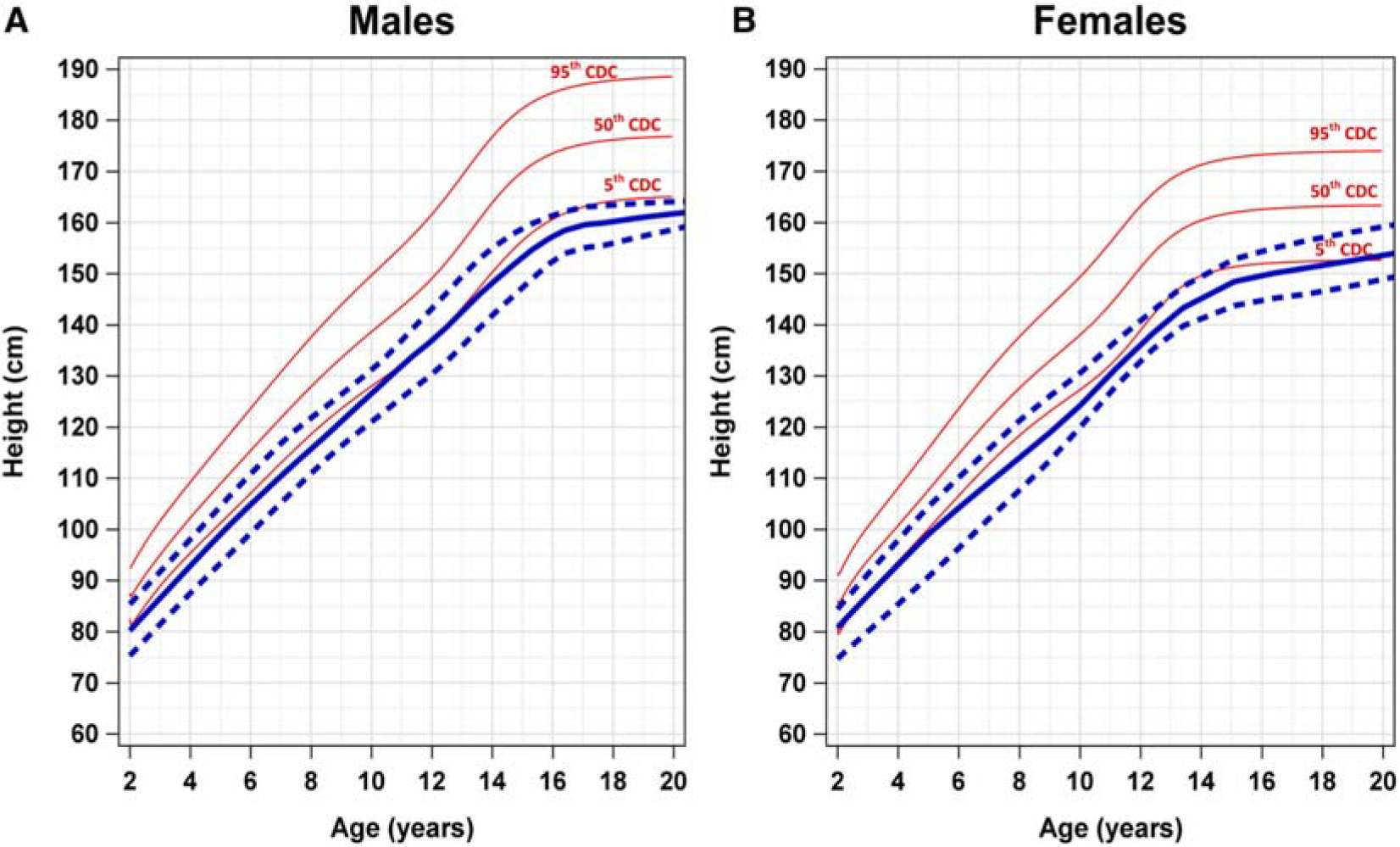
Comparison of height-for-age growth curves of male and female patients with SDS and CDC references for ages 2–20 years. Comparison of height-for-age growth curves of (**A**) male and (**B**) female patients with SDS and CDC percentiles (*red*) for ages 2–20 years. The *solid blue line* represents the 50th percentile SDS height-for-age growth curve. The *blue dashed lines* represent the 95th percentile (*top*) and the fifth percentile (*bottom*) height-for-age growth curves. *CDC*, Centers for Disease Control and Prevention; *SDS*, Shwachman-Diamond syndrome.

**Figure 2.**
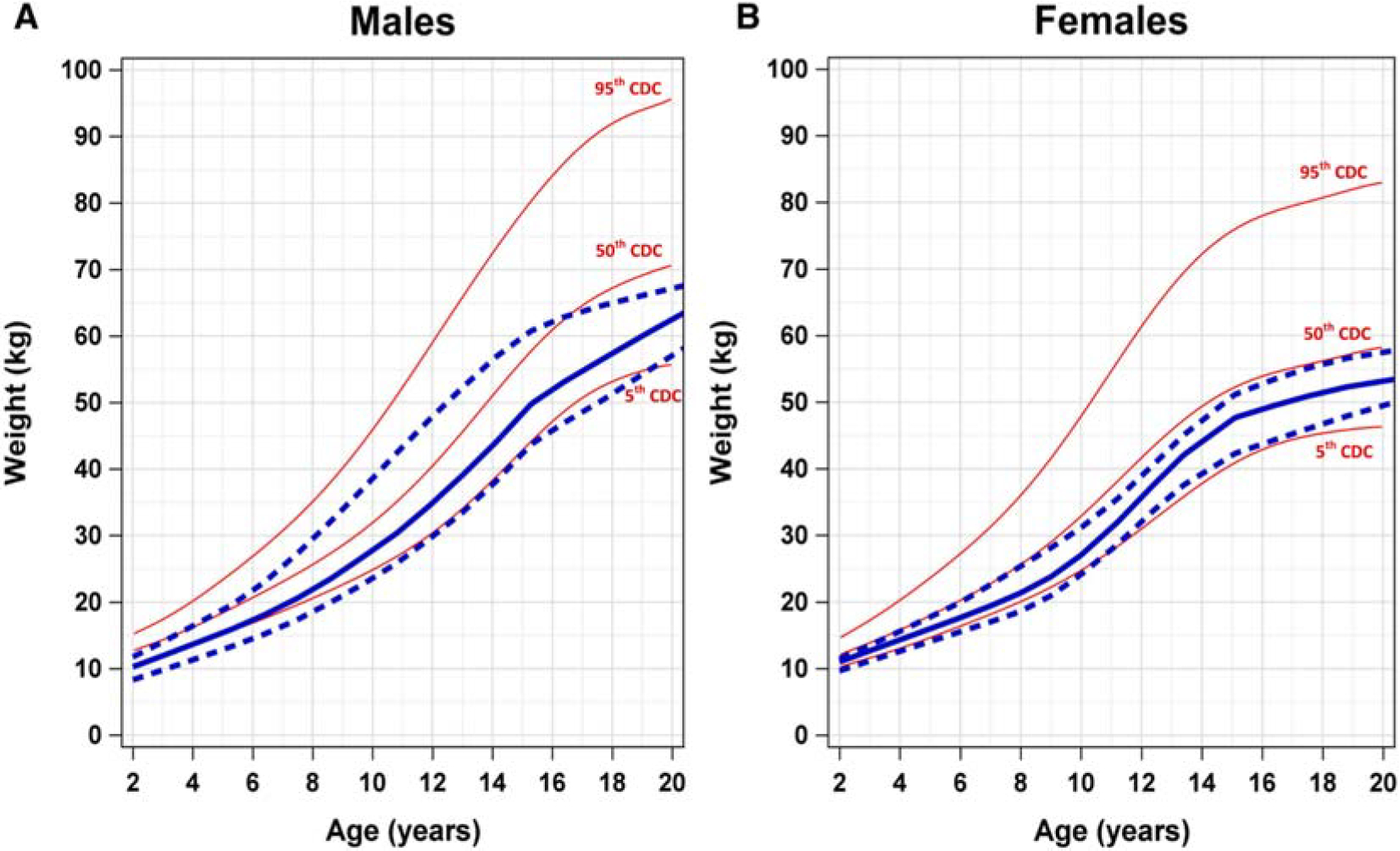
Comparison of weight-for-age growth curves of male and female patients with SDS and CDC references for ages 2–20 years. Comparison of weight-for-age growth curves of (**A**) male and (**B**) female patients with SDS and CDC percentiles (*red*) for ages 2–20 years. The *solid blue line* represents the 50th percentile SDS weight-for-age growth curve. The *blue dashed lines* represent the 95th percentile (*top*) and the fifth percentile (*bottom*) weight-for-age growth curves. *CDC*, Centers for Disease Control and Prevention; *SDS*, Shwachman-Diamond syndrome.

**Figure 3.**
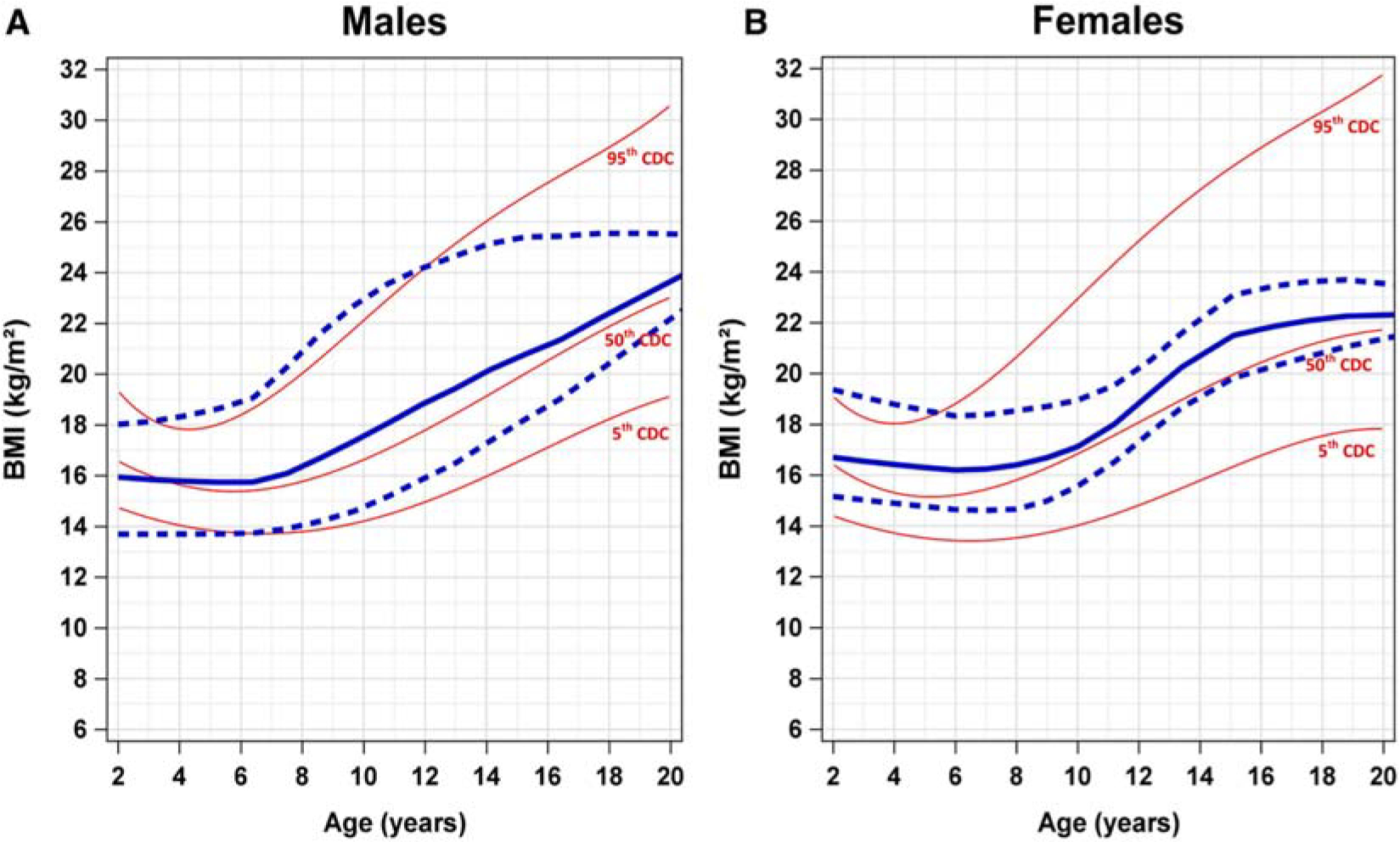
Comparison of BMI-for-age growth curves of male and female patients with SDS and CDC references for ages 2–20 years. Comparison of BMI-for-age growth curves of (**A**) male and (**B**) female patients with SDS and CDC percentiles (*red*) for ages 2–20 years. The *solid blue line* represents the 50th percentile SDS BMI-for-age growth curve. The *blue dashed lines* represent the 95th percentile (*top*) and the fifth percentile (*bottom*) BMI-for-age growth curves. *BMI*, body mass index; *CDC*, Centers for Disease Control and Prevention; *SDS*, Shwachman-Diamond syndrome.

We generated SDS-specific growth curves for both males and females superimposed over established CDC reference curves (Figures 1–3). Due to the potential impact of HSCT procedures and exposures and GH supplementation, these growth curves only include values prior to HSCT or initiation of GH.

Height-for-age and weight-for-age growth patterns were below the reference curves for both male and female patients aged 2–20 years with SDS. Comparison of height-for-age curves of patients with SDS shows the 50th percentile for both males and females corresponded to the fifth percentile CDC reference ranges (Figure 1). This trend is maintained through the observation period, up to 20 years.

A similar pattern is also observed for the weight-for-age growth curves in male and female patients aged 2–20 years with SDS. Comparison of weight-for-age curves of patients with SDS shows the 50th percentile tracks slightly above the fifth percentile but below the 50th percentile CDC reference ranges (Figure 2).

Interestingly, BMI-for-age growth curves in both male and female patients with SDS were comparable with CDC reference curves (Figure 3). In both male and female patients, the 50th percentile of BMI-for-age growth curves was similar to the 50th percentile of the CDC reference ranges. Furthermore, the 95th percentile BMI-for-age growth pattern in male patients with SDS was similar to the 95th percentile CDC reference range but fell below around age 13 years. However, the 95th percentile BMI-for-age growth curve in female patients with SDS started out above the corresponding CDC 95th percentile reference curve in early years, BMI-for-age fell below this reference curve after age 6 years and never caught up by the end of the observation period.

### SDS Growth Curves for Ages 0–2 Years

**Figure 4.**
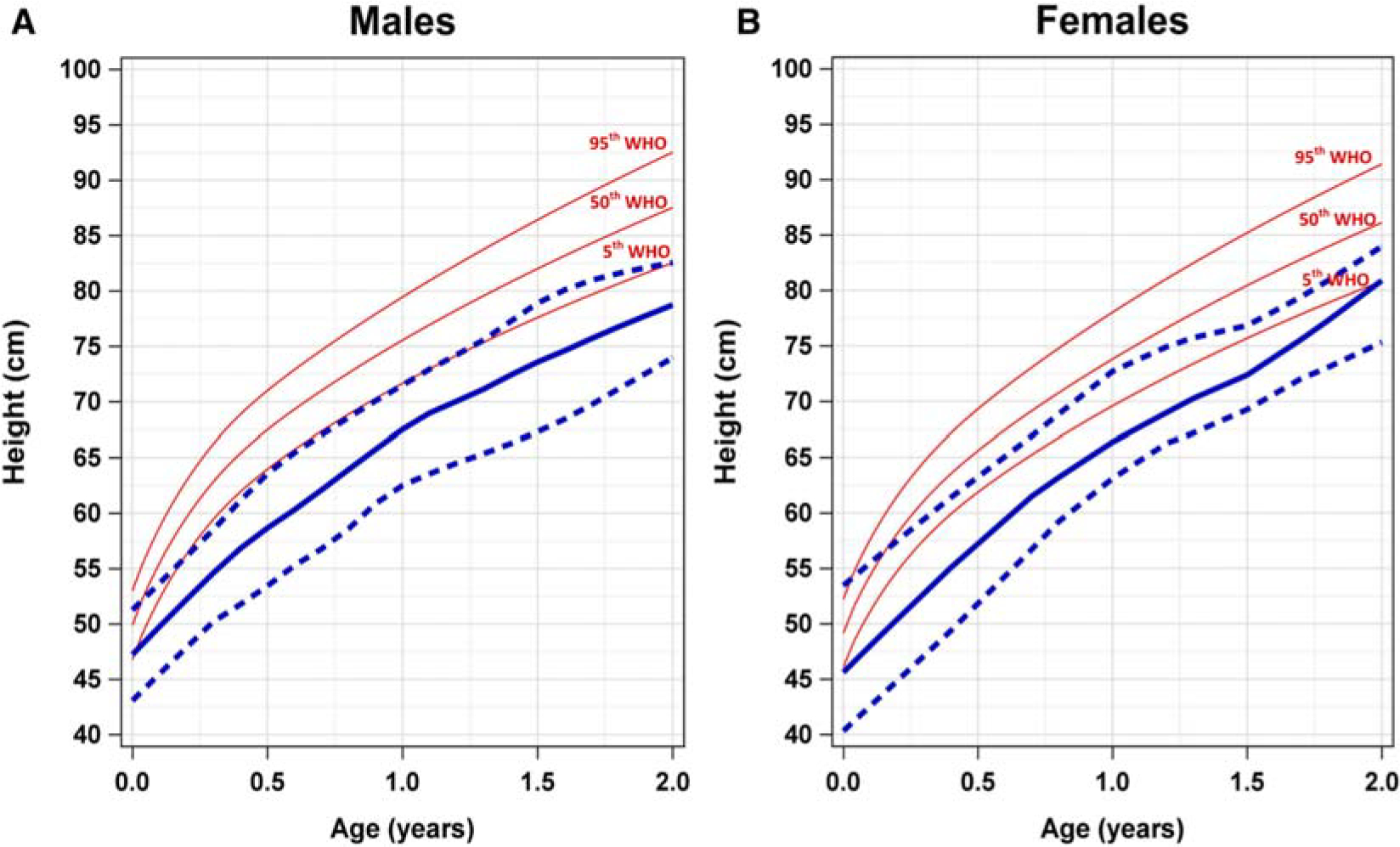
Comparison of height-for-age growth curves of male and female patients with SDS and WHO references for ages 0–2 years. Comparison of height-for-age growth curves of (**A**) male and (**B**) female patients with SDS and WHO percentiles (*red*) for ages 0–2 years. The *solid blue line* represents the 50th percentile SDS height-for-age growth curve. The *blue dashed lines* represent the 95th percentile (*top*) and the fifth percentile (*bottom*) height-for-age growth curves. *SDS*, Shwachman-Diamond syndrome; *WHO*, World Health Organization.

We also generated height-for-age and weight-for-age growth patterns for male and female patients aged between 0 and 2 years with SDS and overlaid them with WHO reference curves. We observed similar growth patterns for height and weight in this age group compared with patients aged 2–20 years old. Comparison of height-for-age curves of patients with SDS shows the 50th percentile for both males and females fell below the fifth percentile WHO reference ranges (Figure 4). This trend was maintained through the observation period, up to 2 years. For male patients specifically, the 95th percentile height-for-age growth curve corresponded with the fifth percentile WHO reference curve. However, for female patients, the 95th percentile height-for-age corresponded between the 5th and 50th percentile WHO reference curve.

**Figure 5.**
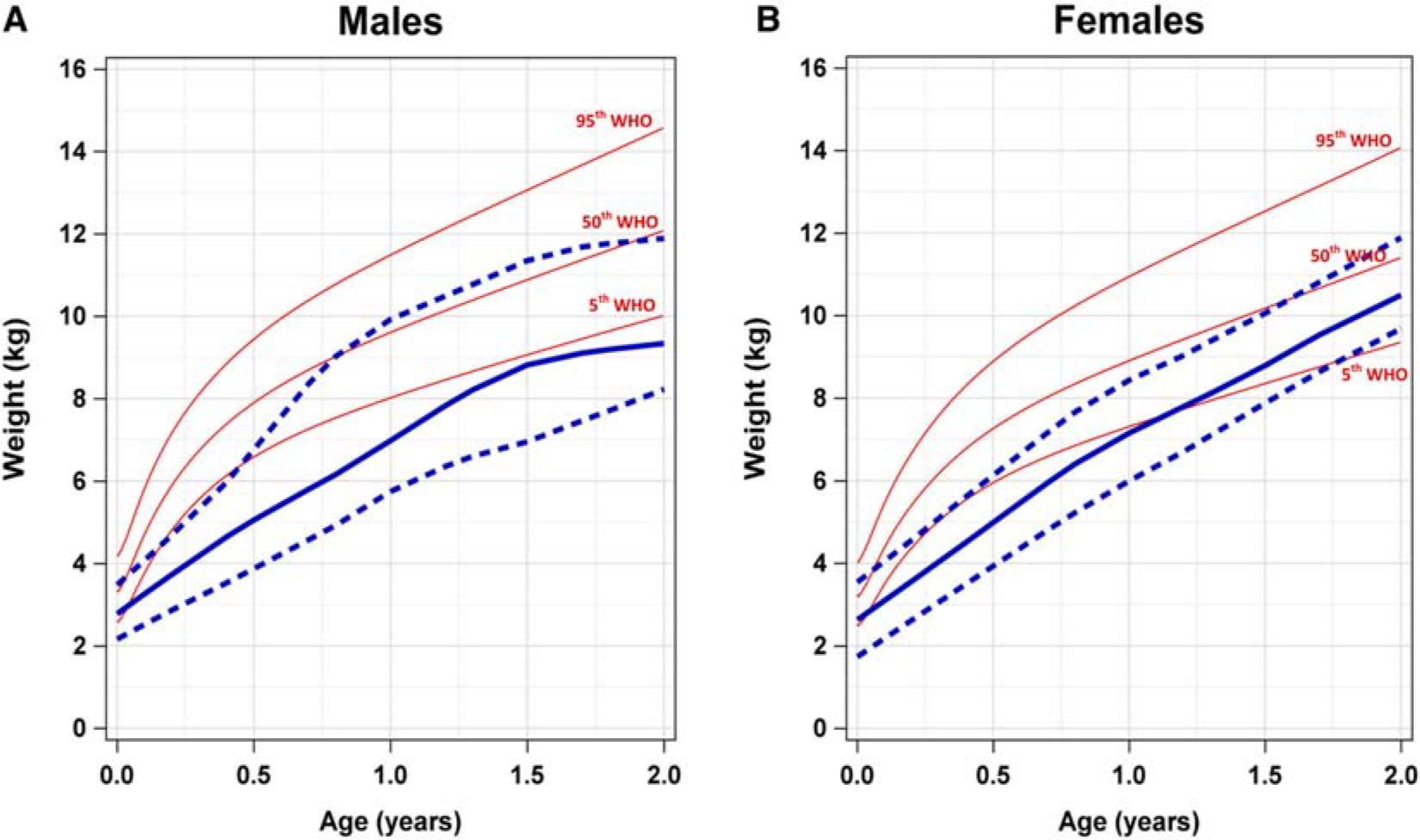
Comparison of weight-for-age growth curves of male and female SDS patients and WHO references for ages 0–2 years. Comparison of weight-for-age growth curves of (**A**) male and (**B**) female patients with SDS and WHO percentiles (*red*) for ages 0–2 years. The *solid blue line* represents the 50th percentile SDS weight-for-age growth curve. The *blue dashed lines* represent the 95th percentile (*top*) and the fifth percentile (*bottom*) weight-for-age growth curves. *SDS*, Shwachman-Diamond syndrome; *WHO*, World Health Organization.

Weight-for-age growth curves in male and female patients with SDS are shown in Figure 5. Our data demonstrate that for males with SDS, the 50th percentile weight-for-age growth curve was below the fifth percentile WHO reference curve. In female patients with SDS, the 50th percentile weight-for-age growth curve begins below the fifth percentile, by around 1 year of age this curve crosses the fifth percentile WHO reference curve and continues to rise by the end of the observation period. For both male and female patients, the 95th percentile weight-for-age growth curves overlaps closely with the 50th percentile WHO reference population.

The height-for-age, weight-for-age, and BMI-for-age Z-scores in patients with SDS by each year are reported in Supplementary Table 1; available at www.jpeds.com.

### Comparison of Projected Height, Weight, and BMI at Different Timepoints of Growth

Table II presents a numerical comparison of estimated attained height, weight, and BMI at various growth milestones (ages 4, 8, 12, 16, and 20 years) between patients with SDS and CDC reference data, stratified by gender. Notably, the median adult (aged 20 years) height of individuals with SDS was lower than CDC standards, with females and males measuring approximately 7 cm and 11 cm shorter. Across all age groups, both male and female patients with SDS exhibited lower median height compared with CDC references. This trend was also observed for weight in SDS females but not in SDS males. Conversely, BMI estimates for SDS males and females were comparable with CDC reference values.

### Effect of HCST on Height Trajectory

**Figure 6.**
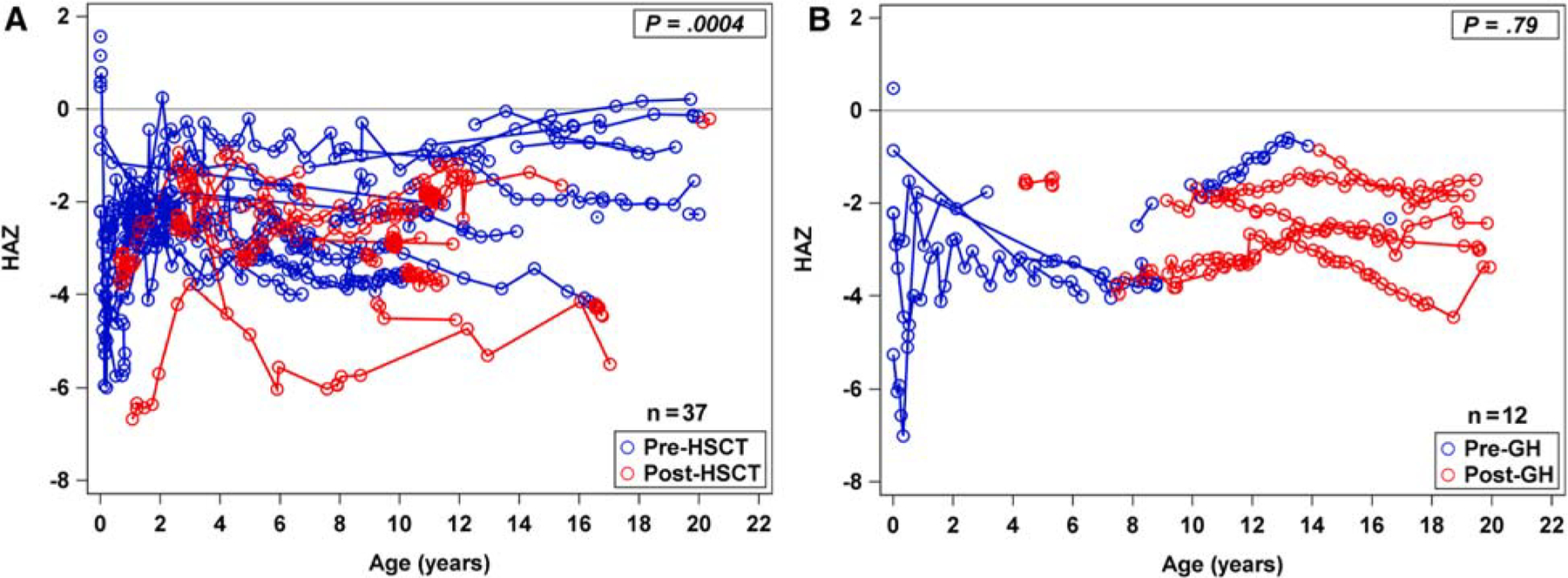
Comparison of height-for-age Z-scores of all SDS patients who received hematopoietic stem cell transplant. (**A**) Comparison of height-for-age Z-scores (HAZ) of patients with SDS who received HSCT excluding any height measurements after start of GH therapy (n = 36). The *blue dots/lines* represent all height observations prior to HSCT (pre-bone marrow transplant). The *red dots/lines* represent all height observations after HSCT (post-bone marrow transplant). Connected lines represent the same subject. There was a significant decrease in HAZ slope after HSCT compared with pre-HSCT (slope change post-HSCT = −0.26, *P* = .0004). (**B**) Comparison of height-for-age Z-scores (HAZ) of all patients with SDS who received GH treatment (n = 12) excluding any height measurements after HSCT. The *blue dots/lines* represent all height observations prior to GH (pre-GH). The *red dots/lines* represent all height observations after GH (post-GH). After starting GH, there was no significant change in HAZ slope compared with pre-GH (slope change post-GH = 0.04; *P* = .79). *GH*, growth hormone; *HSCT*, hematopoietic stem cell transplant; *SDS*, Shwachman-Diamond syndrome.

Since about one-third of our patient cohort underwent HSCT, we examined how exposure to transplantation procedures influenced HAZ scores post-HSCT. Figure 6A compares the HAZ scores for patients before and after HSCT. Any measurements after GH initiation were excluded for this analysis to prevent confounding (36 patients included with 637 observations). There was a significant decrease in the HAZ slope after HSCT compared with pre-HSCT (slope change post-HSCT = −0.26, *P* = .0004).

### Effect of GH Replacement Therapy on Height Trajectory

Although only 12 patients (10%) received GH therapy, this limits our analysis to small numbers; we characterized the HAZ before and after GH therapy for these patients. In this subset of patients, GH therapy was initiated at a median of 9.3 years (range: 4.4–17 years) and duration of therapy lasted for a median of 4 years (range: 0.9–11 years). To limit potential confounding, we excluded any observations after HSCT for this analysis (12 patients still included with 269 observations). After starting GH therapy, there was no significant change in the HAZ slope compared with pre-GH (slope change post-GH = 0.04, *P* = .79) (Figure 6B). No patients developed myelodysplastic syndrome or acute myeloid leukemia due to GH therapy.

## Discussion

Our study characterizes growth patterns in patients with SDS from North America. Our data support and extend findings from the Italian SDS Registry and align with prior observations that growth parameters appear similar between the Italian and the North American cohorts.

Consistent with prior studies, our findings confirm that individuals with SDS exhibit significant growth impairments compared with CDC and WHO reference populations despite pancreatic enzyme therapy for patients with exocrine pancreatic insufficiency. Across all age groups, both males and females with SDS consistently demonstrated lower HAZ, with the median adult height being approximately 7 cm and 12 cm shorter than CDC standards for females and males, respectively. These findings indicate that differences in growth remain a common clinical feature associated with SDS.

Although weight-for-age patterns were also diminished, BMI-for-age trajectories in SDS patients were comparable with CDC reference values. In contrast to findings reported in the Italian cohort of patients with SDS, where adolescent females demonstrated a disproportionately higher increase in weight and BMI compared with the general population, our cohort exhibited a more uniform pattern of weight and BMI changes across age and sex groups. This finding suggests that individuals with SDS may have relative preservation of weight in proportion to height, a phenomenon that may be influenced by altered body composition or metabolic adaptations. Notably, male patients demonstrated persistently low weight-for-age at the fifth percentile compared with reference populations, whereas female patients initially lagged but eventually reached comparable weight-for-age percentiles in later years.

Given the significant proportion of patients with SDS who undergo HSCT due to progressive bone marrow failure or transformation to myeloid malignancy, the impact of transplantation on growth outcomes is of high clinical interest. Our analysis identified a statistically significant decline in HAZ following HSCT. Our findings are consistent with other studies in other bone marrow failure syndromes, suggesting that HSCT can adversely affect final height due to chemotherapy-related endocrine dysfunction or graft-vs-host disease.^20–22^ However, it is important to note that our cohort included patients transplanted at varying ages and with differing conditioning regimens, which may contribute to individual variability in growth outcomes.

The incidence of GH deficiency is variable, and GH therapy has been proposed as a potential intervention for improving final height outcomes. In our cohort, only a small subset of patients (10%) received GH therapy, limiting our ability to draw definitive conclusions regarding its efficacy. Nevertheless, our analysis did not reveal a significant change in height velocity after GH initiation, even when excluding HSCT recipients. This suggests that although GH may improve short-term growth velocity in some patients with SDS, its long-term impact on final adult height may be limited. This study presents a novel contribution by incorporating growth data from SDS patients who have received GH therapy, a factor not consistently captured in prior reports. Although the number of patients receiving GH therapy in our cohort is limited, our findings suggest only a modest effect of GH on long-term growth outcomes. The limited effect of GH in a skeletal dysplasia such as SDS is not surprising. Thus, these observations may help guide clinical decision-making regarding the use of GH therapy in SDS, particularly in the context of other growth-limiting factors such as HSCT. Further prospective studies with larger sample sizes are needed to assess whether GH therapy provides meaningful benefits in patients with SDS, particularly when initiated earlier in childhood.

Our study builds upon prior important work, such as those by Cipolli et al, to establish SDS-specific growth charts.^12,17^ Their growth curves provided valuable insights into growth patterns in SDS, particularly within an Italian cohort. Our study complements these findings by including a broader age range and a more diverse patient sample, which may enhance the generalizability of the reference standards for clinicians managing SDS. Furthermore, by excluding patients who had undergone HSCT or received GH therapy, we aimed to characterize the natural growth trajectory in SDS without the influence of treatment-related factors. Although prior studies, including those from the Italian SDS Registry, have reported a median age of menarche comparable with the general population,^17^ our registry does not systematically capture pubertal milestones such as age of menarche or pubarche, which limits our ability to assess the potential impact of pubertal timing on growth outcomes—an important consideration when interpreting these findings.

The findings from our study have important implications for the clinical management of patients with SDS. First, our SDS-specific growth curves provide valuable reference points for monitoring growth and identifying deviations that may warrant further endocrine or nutritional evaluation. Second, although our data suggest that HSCT significantly alters height trajectory, careful attention should be paid to long-term endocrine function in transplanted patients to mitigate potential late effects. Finally, the limited impact of GH therapy observed in our cohort underscores the need for further research to identify optimal strategies for improving growth outcomes in SDS, including potential adjunctive therapies beyond GH replacement.

In conclusion, our study provides a more comprehensive understanding of growth patterns in SDS, highlighting persistent short stature and altered weight trajectories in affected individuals. By addressing key knowledge gaps, our findings contribute to the development of more tailored growth monitoring and management strategies for this unique patient population.

## Supplementary Material

supp 1

## Figures and Tables

**Table I.** Cohort study characteristics

Variable	Patient cohort, n = 127
Sex, n (%)	
Male	75 (59)
Female	52 (41)
Race, n (%)	n = 124
White	113 (91)
Black	1 (0.8)
Other	10 (8)
SDS diagnosis age (y), median (IQR)	n = 118
	2.4 (0.6–7.7) [0.4, 39.0]
Birth gestational age ≥ 37 weeks, n (%)	84/107 (78.5)
Birth gestational age ≥ 37 weeks, median	2.9 (2.5–3.3) n = 75
(IQR)	
Birth weight (kg)	19.0 (18.0–19.0) n = 48
Birth length (inches)	2.2 (1.9–2.4) n = 21
Birth gestational age < 37 weeks, median	17.1 (15.4–17.5) n = 14
(IQR)	
Birth weight (kg)	
Birth length (Inches)	
History of pancreatic Insufficiency, n (%)	118/126 (94)
Pancreatic enzyme replacement, n (%)	117/125 (94)
Growth hormone replacement, n (%)	12/123 (10)
Indication for growth hormone, n (%)	5 (42)
Growth hormone deficiency	5 (42)
Short stature	2 (16)
Unknown	
HSCT, n (%)	37 (29)
Indication for HSCT, n (%)	
Acute myeloid leukemia	4 (11)
Myelodysplastic syndrome	10 (27)
Bone marrow failure	14 (38)
High risk features	8 (22)
Unknown	1 (2)
Median age at HSCT in y (IQR)	10.7 (5.7–20.8)
Conditioning regimen, n (%)	
Myeloablative	11 (30)
Reduced Intensity	23 (62)
Unknown	3 (8)
Total body irradiation, n (%)	1 (2.7)
SBDS mutations, n (%)	n = 127
c.258+2T>C and c.183_184TA>CT	78 (61.4)
Homozygous c.258+2T>C	13 (10.2)
c.258+2T>C and c.258+1G>C	6 (4.7)
Homozygous c.258+2T>C and c.183_184TA>CT	4 (3)
Homozygous c.258+2T>C and c.201+A>G	3 (2.3)
c.183_184TA>CT and C.523C>T	1 (0.8)
c.258+2T>C and 616_619del	1 (0.8)
c.258+2T>C and c.107delT	1 (0.8)
c.258+2T>C and c.120delG	2 (1.6)
c.258+2T>C and c.169T>C	1 (0.8)
c.258+2T>C and c.170T>C	1 (0.8)
c.258+2T>C and c.184A>T	1 (0.8)
c.258+2T>C and c.250T>C	2 (1.6)
c.258+2T>C and c.259-2A>G	1 (0.8)
c.258+2T>C and c.25C>T	1 (0.8)
c.258+2T>C and c.355T>C	2 (1.6)
c.258+2T>C and c.41A>G	1 (0.8)
c.258+2T>C and c.458A>G	1 (0.8)
c.258+2T>C and c.460-1G>A	1 (0.8)
c.258+2T>C and c.505C>T	2 (1.6)
c.258+2T>C and c.523delC	2 (1.6)
c.258+2T>C and c.641C>T	1 (0.8)
c.258+2T>C and c.652C>T	1 (0.8)

*HSCT*, hematopoietic stem cell transplant; *IQR*, interquartile range; *SBDS*, Shwachman-Bodian-Diamond syndrome; *SDS*, Shwachman-Diamond syndrome.

Data presented as median (25th-75th percentile) (minimum-maximum) or n (%). Complete data unless otherwise specified by “n = ” or stated denominator.

**Table II.** Comparison of height, weight, and BMI of SDS patients to CDC references by age groups

	Females				Males			
Variable	Height (cm)				Height (cm)			
Age	CDC	SDS	SDS Z-score	*P* value*	CDC	SDS	SDS Z-score	*P* value*
4 years	101.0	94.0	−1.52	.0001	102.5	93.8	−2.21	<.0001
8 years	127.8	114.5	−2.42	.0002	128.1	115.7	−2.23	<.0001
12 years	151.5	135.4	−2.06	.03	149.3	137.8	−1.52	<.0001
16 years	162.6	154.7	−1.23	.008	173.6	159.4	−1.90	.0001
20 years	163.3	156.8	−1.01	.13	176.8	165.0	−1.65	.06
Variable	Weight (kg)				Weight (kg)			
Age	CDC	SDS	SDS Z-score	*P* value*	CDC	SDS	SDS Z-score	*P* value*
4 years	15.8	14.2	−0.79	.0003	16.2	14.0	−1.45	<.0001
8 years	25.6	21.2	−1.33	.002	25.6	21.2	−1.46	<.0001
12 years	41.7	34.9	−0.89	.06	40.5	35.1	−0.81	.03
16 years	53.9	55.5	0.17	.84	60.9	55.5	−0.71	.01
20 years	58.2	53.3	−0.56	.25	70.6	69.8	−0.07	.44
Variable	BMI (kg/m^2^)				BMI (kg/m^2^)			
Age	CDC	SDS	SDS Z-score	*P* value*	CDC	SDS	SDS Z-score	*P* value*
4 years	15.3	16.2	0.63	.02	15.6	15.8	0.06	.59
8 years	15.8	16.1	0.16	.54	15.8	15.8	0.04	.76
12 years	18.0	19.0	0.33	.56	17.8	18.9	0.44	.17
16 years	20.4	23.9	0.91	.25	20.5	21.0	0.16	1.00
20 years	21.7	23.2	0.41	.38	23.0	25.3	0.64	1.00

*BMI*, body mass index; *CDC*, Centers for Disease Control and Prevention; *SDS*, Shwachman-Diamond syndrome.

Data presented as 50th percentile for CDC and medians for SDS.

* *P* values testing whether SDS Z-score differed from 0 (CDC population normal).

## Data Availability

Data sharing statement available at www.jpeds.com.

## Abbreviations

BMI: Body mass index
CDC: Centers for Disease Control and Prevention
GH: Growth hormone
HAZ: Height-for-age Z-scores
HSCT: Hematopoietic stem cell transplant
IQR: Interquartile range
SDS: Shwachman-Diamond syndrome
WHO: World Health Organization
