# Supplementary material for: Growth Patterns in Shwachman-Diamond Syndrome: Findings from the North American Shwachman-Diamond Syndrome Registry
Source: J Pediatr. Author manuscript; Available in PMC 2026 Jun 8. (PMC13244479; doi:10.1016/j.jpeds.2025.114780)
Supplement: supp 1 [file NIHMS2170322-supplement-supp_1.docx]

**Supplementary Table 1. Height, weight and BMI-for- age Z-scores in patients with SDS at all age groups**

| Age* | Age (years) | Height-for-age  Z-score (HAZ) | Weight-for-age  Z-score (WAZ) | BMI Z-score |
| --- | --- | --- | --- | --- |
| Term birth (n=75)  [≥ 37 weeks] | 0.0 (0.0, 0.0) | *n=48*  -0.86 (-2.21, -0.16) | -0.84 (-1.79, -0.12) | *n=48*  -0.59 (-1.86, 0.35) |
| 0> - <1 year (n=44) | 0.36 (0.13, 0.65) | -3.41 (-4.85, -1.84) | -3.15 (-4.12, -1.80) | -1.70 (-2.49, -0.65) |
| 1 - <2 years (n=47) | 1.22 (1.03, 1.42) | -3.18 (-3.70, -2.30) | -2.02 (-3.08, -1.18) | -0.35 (-1.06, 0.75) |
| 2 - <3 years (n=40) | 2.18 (2.05, 2.40) | -2.12 (-2.78, -1.34) | -1.57 (-2.51, -0.65) | 0.19 (-0.65, 0.72) |
| 3 - <4 years (n=45) | 3.20 (3.08, 3.42) | -1.92 (-2.57, -1.28) | -1.18 (-2.08, -0.60) | 0.11 (-0.57, 0.92) |
| 4 - <5 years (n=43) | 4.18 (4.08, 4.39) | -1.98 (-2.45, -1.24) | -1.07 (-2.05, -0.60) | 0.33 (-0.73, 0.95) |
| 5 - <6 years (n=39) | 5.20 (5.14, 5.43) | -2.15 (-2.60, -1.59) | -1.53 (-2.39, -0.85) | 0.17 (-0.79, 0.78) |
| 6 - <7 years (n=44) | 6.22 (6.09, 6.52) | -2.04 (-2.56, -1.25) | -1.37 (-2.31, -0.72) | 0.05 (-0.87, 0.87) |
| 7 - <8 years (n=32) | 7.25 (7.12, 7.62) | -2.10 (-2.66, -1.30) | -1.39 (-1.88, -0.73) | 0.18 (-0.72, 0.73) |
| 8 - <9 years (n=43) | 8.24 (8.13, 8.52) | -2.05 (-2.68, -1.37) | -1.32 (-2.04, -0.68) | 0.00 (-0.87, 0.86) |
| 9 - <10 years (n=43) | 9.25 (9.11, 9.60) | -1.96 (-2.44, -1.10) | -0.91 (-1.82, -0.19) | 0.13 (-0.53, 0.97) |
| 10 - <11 years (n=40) | 10.2 (10.1, 10.5) | -1.80 (-2.33, -1.19) | -0.95 (-1.93, 0.02) | 0.24 (-0.54, 1.30) |
| 11 - <12 years (n=35) | 11.3 (11.1, 11.5) | -1.63 (-2.09, -1.04) | -0.76 (-1.42, 0.06) | 0.31 (-0.22, 1.20) |
| 12 - <13 years (n=31) | 12.2 (12.1, 12.6) | -1.69 (-2.18, -1.00) | -1.06 (-1.58, -0.25) | 0.03 (-0.92, 0.90) |
| 13 - <14 years (n=28) | 13.3 (13.1, 13.6) | -1.35 (-2.51, -0.89) | -0.37 (-1.95, 0.03) | 0.44 (-0.63, 0.88) |
| 14 - <15 years (n=22) | 14.4 (14.2, 14.7) | -1.90 (-2.91, -1.07) | -0.89 (-1.94, 0.35) | 0.35 (-1.41, 1.06) |
| 15 - <16 years (n=24) | 15.3 (15.2, 15.6) | -1.49 (-2.18, -1.11) | -0.76 (-1.85, 0.05) | 0.08 (-1.39, 0.81) |
| 16 - <17 years (n=26) | 16.4 (16.2, 16.6) | -1.75 (-2.02, -1.15) | -0.55 (-1.73, 0.50) | 0.38 (-0.63, 1.37) |
| 17 - <18 years (n=19) | 17.3 (17.2, 17.5) | -1.48 (-2.18, -0.85) | -0.41 (-1.78, 0.20) | 0.10 (-1.00, 1.39) |
| 18 - <19 years (n=17) | 18.2 (18.1, 18.4) | -1.31 (-2.03, -0.49) | -0.91 (-2.33, -0.44) | -0.34 (-1.51, 0.23) |
| 19 - <20 years (n=17) | 19.3 (19.2, 19.5) | -1.53 (-2.26, -0.75) | -0.64 (-2.29, 0.27) | 0.00 (-1.23, 0.66) |

Data presented as median (25^th^-75^th^ percentile).

*First observation used per subject within age range (subjects only represented once in each age range)
